# Building a Cell and Anatomy Ontology of *Caenorhabditis Elegans*

**DOI:** 10.1002/cfg.248

**Published:** 2003-02

**Authors:** Raymond Y. N. Lee, Paul W. Sternberg

**Affiliations:** WormBase 156-29 California Institute of Technology Pasadena CA 91125 USA

## Abstract

We are endowed with a rich knowledge about *Caenorhabditis elegans*. Its stereotyped anatomy and development has stimulated research and resulted in the accumulation of cell-based information concerning gene expression, and the role of specific cells in developmental signalling and behavioural circuits. To make the information more accessible to sophisticated queries and automated retrieval systems, WormBase has begun to construct a *C. elegans cell* and anatomy ontology. Here we present our strategies and progress.


**Comparative and Functional Genomics**
*Comp Funct Genom* 2003; **4**: 121–126.
Published online in Wiley InterScience (www.interscience.wiley.com). **DOI:** 10.1002/cfg.248

**Conference Review**

### Building a cell and anatomy ontology of *Caenorhabditis elegans*

Raymond Y. N. Lee* and Paul W. Sternberg
*WormBase, Division of Biology, California Institute of Technology, Pasadena, CA 91125, USA*

*Correspondence to:
Raymond Y. N. Lee, WormBase, 156-29 California Institute of Technology, Pasadena, CA 91125, USA.
E-mail: raymond@caltech.edu
URL: http://wormbase.org

Received: 11 November 2002
Accepted: 2 December 2002

### Abstract

**We are endowed with a rich knowledge about *Caenorhabditis elegans*. Its stereotyped anatomy and development has stimulated research and resulted in the accumulation of cell-based information concerning gene expression, and the role of specific cells in developmental signalling and behavioural circuits. To make the information more accessible to sophisticated queries and automated retrieval systems, WormBase has begun to construct a *C. elegans cell* and anatomy ontology. Here we present our strategies and progress. Copyright © 2003 John Wiley & Sons, Ltd.**

**Keywords:** ***Caenorhabditis elegans*; ontology; cell and anatomy; information technology**

### Introduction

Ontologies allow better organization of knowledge. By explicitly specifying semantics and relations, ontologies make it possible to effectively organize complex information. A successful ontology consists of factual statements organized in a strictly logical manner. Consequently, an ontology can be expressed in a computer-understandable language, dramatically increasing its utility.

Ontologies are useful to biologists; the great success of the Gene Ontology (GO; **http://www.geneontology.org**) is evidence of this. By organizing accumulated knowledge about biology into three orthogonal ontologies, GO provides a summary of what we know in a logical, machine-readable structure. GO has allowed improved interpretation of large-scale genomic analysis. The usefulness of ontologies goes up dramatically as the amount and the degree of complexity of biological knowledge increases.

There is detailed information on many aspects of the cells and anatomy of *Caenorhabditis elegans*: we know how many cells there are in a *C. elegans* at all times of its life cycle [4,5,7,10–13]; we have a near-precise knowledge of most cells' lineage and developmental fates, and in a few cases the nature of developmental indeterminacy; we know, at the electron microscopy (EM) level, how most neurons are connected to each other [1,15]; we know the function of many cells in development or in mature animals; and we have detailed functional and morphological information on anatomy (generally reviewed in [6,16]). Thus, a *C. elegans* cell and anatomy ontology (CECAO) would be useful to organize all this information.

### *C. elegans* cells and anatomy

*C. elegans* is a free-living soil nematode that feeds on bacteria in the laboratory. It is small (about 1 mm long and 80 μm in diameter when fully grown) and semitransparent. It has two sexes, self-fertilizing hermaphrodite (morphologically female) and male. *C. elegans* has seven major developmental stages: embryo, four larval stages (L1–L4), reproductive adult, and a dispersal dauer larval stage that is alternative to L3 and takes place only under certain harsh living conditions.

As for other nematodes, *C. elegans* has a general body plan that is made up of two concentric tubes separated by a pseudocoelom. The outer tube consists of a single cell layer epidermis,

Copyright © 2003 John Wiley & Sons, Ltd.

four body-wall muscle quadrants, and neurons. The inner tube contains the alimentary system, comprising (in anterior to posterior order) the pharynx, intestine and hindgut. Adults also have gonads in the pseudocoelomic space. The most complex organ system in the worm is the nervous system, which is organized in several ganglions. Neurons make contacts *en passant* and the major nerve bundles form the circumpharyngeal nerve ring and the dorsal and ventral cord (reviewed by [16]).

Because of its transparency, one can observe the process of *C. elegans* development *in vivo* with the aid of differential interference contrast (Nomarski) optics. Thus, Sulston *et al.* [13] painstakingly traced the full embryonic cell lineage, from single-cell zygotes to either 558-cell hermaphrodite or 560-cell male hatchlings. During embryogenesis, cells divide, migrate, differentiate and sometimes die. Confirming what others had noted (reviewed by [2]), Sulston *et al.* [13] found that the nematode follows a largely invariant cell lineage pattern, an important feature that enables studying of the process of development via lineage analysis.

During post-embryonic development, somatic blast cells continue to divide and differentiate through larval stages, so that a mature hermaphrodite has 959 somatic nuclei, whereas an adult male has 1031 [16]. Post-embryonic cell lineages have been traced by Sulston and Horvitz (focus on somatic cells [12]) and by Kimble and Hirsh (focus on gonads [4]). Although most cell fates are rigid and invariable, there are a few exceptions, e.g. in the hermaphrodite lineages that ultimately form the somatic gonad, there are two alternative lineages involving the blast cells Z1.ppa, Z1.ppp, Z4.aaa, and Z4.aap (cells are named by their lineage; a, anterior; p, posterior; such that Z1.p is the posterior daughter of Z1). The two alternative lineage patterns are related by a two-fold rotational symmetry ([4]).

Although useful in lineage tracing experiments, Nomarski optics is insufficient in working out cell–cell contacts and some subcellular details. Using EM and reconstruction of serial thin sections, fine anatomical and cellular details have been delineated for many parts of the worm, including the anterior sensory organ [14], the pharynx [1], and the male sexual organs [11]. A particularly heroic body of work is the reconstruction of the entire nervous system from electron micrographs by White *et al.* [15], which provides an anatomical sketch of how neurons are connected to each other, to muscle and to other postsynaptic partner cells. Based on the anatomical analyses, neurons are grouped into classes, such that members of each class share similar anatomy and thus may also perform similar functions. Using the green fluorescent protein (GFP) labelling technique, one can now also observe subcellular anatomical features in live animals by light microscopy.

*C. elegans* researchers have been taking advantage of this deep knowledge of cells as the basis for experimental analyses. Consequently, *C. elegans* research is very much rooted in the knowledge of cells and anatomy, e.g. gene expression is routinely annotated to specific cells in addition to tissues; specific effects on cell lineages and fates are analysed for genetic and physical manipulations (such as laser microsurgery); and proposed neuronal pathways are tested for their roles in mediating specific animal behaviours.

### Design of CECAO

The objective of CECAO is to provide an ontology that contains all the information about *C. elegans* cell and anatomy so that the information can be parsed by computer programs. However, we would also like an ontology of controlled vocabularies that readily supports annotation of experimental results, such that the outcomes can be queried effectively, e.g. we would like the ontology to support complex queries such as, 'Which genes are expressed in the lineage parents of pharyngeal, but not of somatic sensory, neurons?', or 'Which cholinergic neurons are in the tail region of the male?'

Our ontology will consider five major aspects: cell lineage, position, cell type, organ and function. A cell can be identified by one or more of these aspects, e.g. a cell whose formation follows the lineage AB.plpaappaa, has its nucleus in the left lateral ganglion, is a neuron, is part of the amphid sensilla, and senses touch, high levels of osmolarity and other forms of noxious stimuli, is ASHL. The complex nature of this range of information precludes a simple, hierarchical tree format; instead, a more complex data structure is needed, i.e. a directed acyclic graph.

Copyright © 2003 John Wiley & Sons, Ltd. 

As we began to build this CECAO, we realized that we needed to apply new strategies to achieve logical consistency and to be able to represent all knowledge of *C. elegans* cells and anatomy. Here we discuss a few examples.

### The distinction between a cell and its nucleus

- *Lineage*: Although usually referred to as the 'cell lineage', a lineage determined by observation with Nomarski optics primarily concerns nuclei. A nucleus divides to give rise to two nuclei. Whereas the identity of a cell is often established by a set of properties, a nucleus has a defined parentage and thus a precise position in a lineage. Therefore, CECAO uses nuclei to define nodes in lineages. A child nucleus has a DESCENDENT_OF relationship with the parent nucleus.
- *Syncytia*: Like other metazoans, some cells in the worm are syncytia. A syncytium is usually either the product of cell fusions or incomplete cell divisions that result in a cell with multiple nuclei. Thus, in CECAO, for each nucleus we define a node and it has a PART_OF relationship with the syncytium that contains it.

### Sexual dimorphism

*C. elegans* has two sexes: hermaphrodite and male. Although the two sexes develop almost identically during embryogenesis, they differ substantially in larval development. To encode these differences in lineages, we apply two new relationships, DESC_MALE (descendent of in male only) and DESC_HERM (descendent of in hermaphrodite only), e.g. the nucleus AB.prpppaaaa becomes a blast cell nucleus Y only in a male, and with 10 cell divisions generates the cells of the two post-cloacal sensilla. Thus, we denote the division of Y with two statements 'Y DESC_MALE Y.a' and 'Y DESC_MALE Y.b'.

### Indeterminacy

Not all cells in *C. elegans* have a precisely predetermined fate, e.g. in the middle of the L1 stage, precursors of the P blast cells migrate from left and right ventrolateral positions into the ventral cord. In most cases the migration within each pair is stochastic and their anterior–posterior positions in the ventral cord determine their cell fates, e.g. for each of the P1 and P2 cells, it cannot be known *a priori* whether it would be derived from the left (AB.plapaapp) or the right (AB.prapaapp) precursor ([10]). For this process, we create a node to represent the indeterminate state P1/P2 and include four statements: 'P1/P2 DEVELOPS_FROM AB.plapaapp'; 'P1/P2 DEVELOPS_FROM AB.prpppaaaa'; 'P1 DEVELOPS_FROM P1/P2'; and 'P2 DEVELOPS_FROM P1/P2'.

A more dramatic case of alternate lineages is found during hermaphrodite somatic gonad development. Four cells, Z1.ppa, Z1.ppp, Z4.aaa and Z4.aap, acting as a group, follow either of two configurations that are termed '5R' and '5L' ([4]; Figure 1a). To represent this process (Figure 1b), we create yet another developmental state, Z1.ppx/Z4.aax. We state that Z1.ppx/ZZ4.aax DEVELOPS_FROM each of the four precursors. The node Z1.ppx/Z4.aax represents an uncommitted developmental state. Furthermore, we incorporate two partially committed states, Z1.ppx/Z4.aax(5R) and Z1.ppx/Z4.aax(5L), each having a DEVELOPS_FROM relationship with Z1.ppx/Z4.aax. Emerging from these partially committed states are invariable, committed lineages, e.g. Z1ppx/Z4.aax(5R) is the parent node of Z1.ppp(5R), which in turn is the parent node of the anchor cell (AC) nucleus. The node 'AC nucleus' has a PART_OF relationship with the node 'anchor cell'.

### Current progress and future plans

We have been constructing CECAO using the DAG-Edit tool provided by GO (**http://sourceforge.net/project/showfiles.php?group_id = 36855**). We have imported sets of data from available resources (Anatace, developed by Sylvia Martinelli; and the 'parts list' provided by Leon Avery; personal communications) into DAG-Edit and manually reorganized the nodes by applying rules such as those mentioned above. We currently have an ontology with about 5000 nodes, one-third of which have definitions. We do not yet know precisely how many nodes there will be in the

Copyright © 2003 John Wiley & Sons, Ltd.

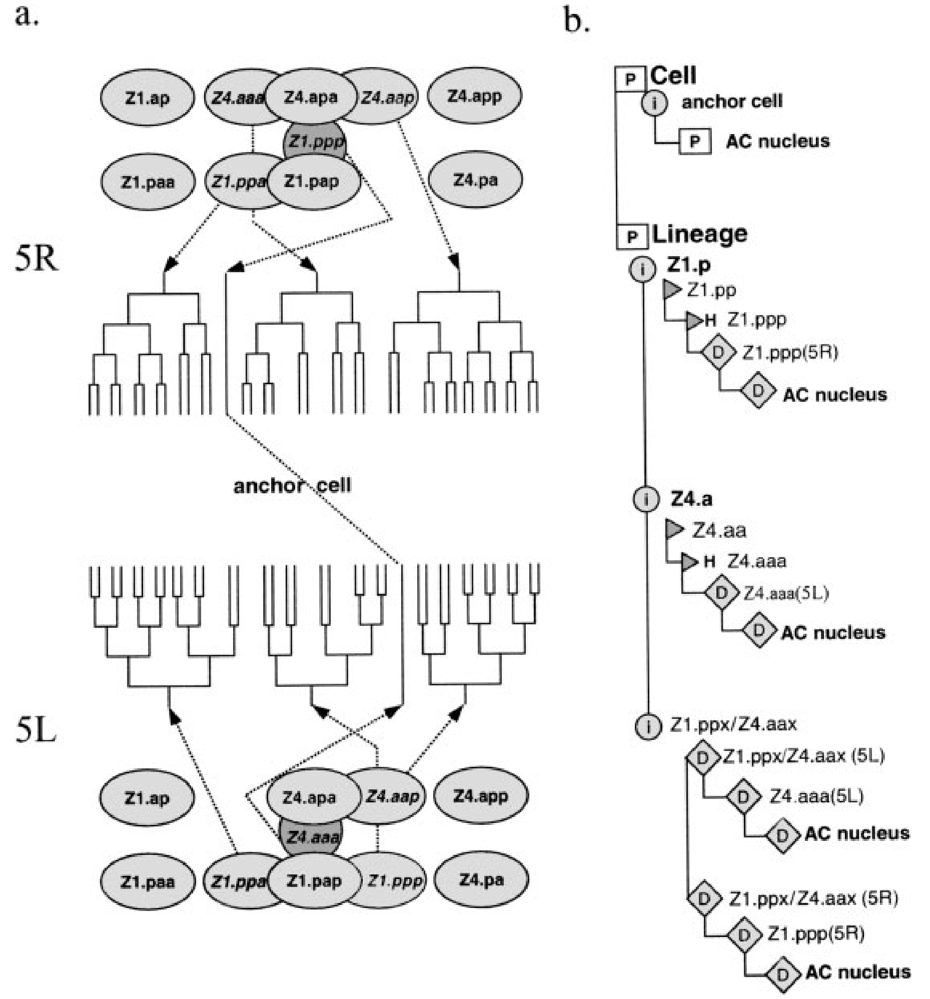

**Figure 1.** Schematic of a *C. elegans* lineage indeterminacy that occurs during the development of hermaphrodite somatic gonads (described by [4]), and its representation in the CECAO ontology. (a) Partial and simplified depictions of two alternative developmental lineage patterns (5R and 5L) that occur in a *C. elegans* hermaphrodite (described in detail by [4]). Ovals in the top and bottom parts represent two possible arrangements of nuclei that may be found in the somatic gonad primordium of a young animal. During larval development, one of the two arrangements, 5R (top) and 5L (bottom), takes place. This process is stochastic. However, once one pattern of nuclear and cell arrangement forms, the developmental process that ensues is fully determined, following either the 5R or 5L lineage pattern (represented by two trees with leaf nodes facing each other). Thus, nuclei that are found in the 5R arrangement will all develop according to the 5R lineage pattern as a group, whereas those in the 5L arrangement will all develop according to the 5L lineage pattern as a group. The 5R and 5L patterns are mutually exclusive in an animal and depend on highly reproducible cell–cell interactions. In this way, a full complement of 37 nuclei (in different cells) in the mature animal is ensured, e.g. Z1.ppp does not divide but becomes the nucleus of the anchor cell (AC nucleus) in 5R, whereas it generates 10 progenitors in the 5L lineage pattern. In contrast, Z4.aaa generates 10 nuclei in 5R, but is destined to become part of the anchor cell in 5L. Dotted arrows connect nuclei with their respective lineages. A dashed line connects the leaf nodes that lead to anchor cell in 5R and 5L lineage pattern, respectively. (b) A directed-acyclic graph view of CECAO showing parts of the ontology relevant to the Z1.ppx/Z4.aax lineage indeterminacy depicted in (a), from the perspective of the anchor cell nucleus (AC nucleus). Following from leaf nodes up, the graph shows that the node 'AC nucleus' is part of 'anchor cell' (in the 'Cell' branch) and develops from either 'Z4.aaa(5L)' or 'Z1.ppp(5R)' (in the 'Lineage' branch). 'Z4.aaa(5L)', in turn, develops from 'Z1.ppx/Z4.aax (5L)'. We use the node 'Z1.ppx/Z4.aax' to represent the indeterminate state, and 'Z1.ppx/Z4.aax (5R)' and 'Z1.ppx/Z4.aax (5L)' to represent an 'equivalence group', from which one path will be chosen for further development. 'Z1.ppx/Z4.aax (5R)' and 'Z1.ppx/Z4.aax (5L)' represent states of development corresponding to the 5R and 5L nuclear arrangement shown in (a), respectively. Each of 'Z1.ppx/Z4.aax (5L)' and 'Z1.ppx/Z4.aax (5R)' develops from 'Z1.ppx/Z4.aax', which is a top-level node in the Lineage branch of the ontology. A triangle represents the relationship 'decendent of'; triangle-H, 'decendent of in hermaphrodite only'; D, 'develops from'; P, 'part of'; and I, 'is a'

Copyright © 2003 John Wiley & Sons, Ltd. 

complete ontology. There are currently 3000 cell and 500 cell group terms in WormBase (incorporating data from Anatace). There are also 80 separate lineage trees, with a total of 6000 nodes. Thus, we estimate that CECAO needs to reconcile 15 000 relationships, assuming that each cell, on average, has five edges.

Given the scale of this project, we started by developing an ontology for groups of cells that are of particular immediate use in WormBase. In particular, we have been focusing on supporting the annotation of gene expression and other cell-based experiments, e.g. we have ontologized information about cells in the pharynx, the feeding organ of the worm. From the bottom up, pharyngeal cells are grouped by anatomical location and by cell type, each having multiple layers of complexity (Figure 2). By using the pharynx ontology to annotate gene expression patterns, we will easily be able to support queries such as, 'Which genes are expressed in pharyngeal neurons whose nuclei are in the corpus, but not in the terminal bulb region?'. WormBase already has 2000 gene expression analyses annotated to 1530 cell and cell group terms. These are sufficient samples with which to test our prototype ontologies.

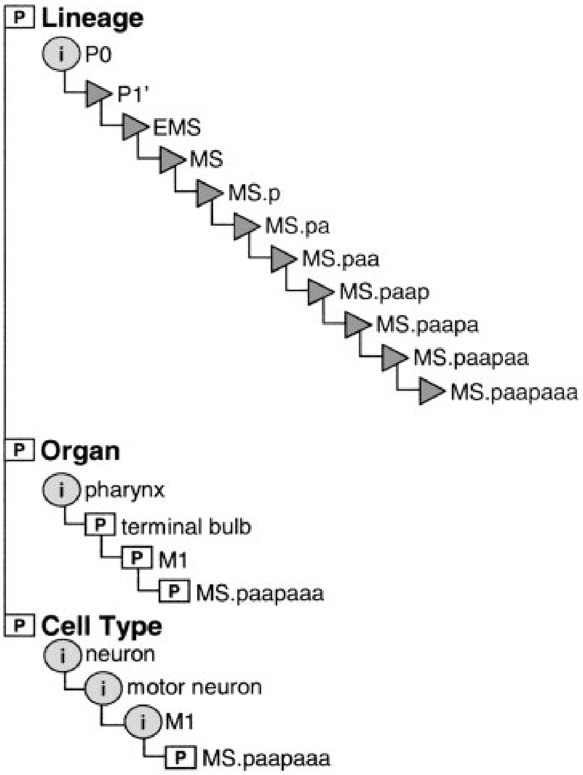

**Figure 2.** A directed-acyclic graph view showing the multiple relations involving the pharyngeal nucleus MS.paapaaa in the CECAO ontology. MS.paapaaa is represented in three major threads: Lineage, Organ and Cell Type. A triangle represents the relationship 'decendent of'; P, 'part of'; and I, 'is a'

One important function of model organism ontologies should be to allow comparisons with ontologies of other organisms; CECAO is currently lacking comparative anatomy. We are collaborating with Worm Atlas (**http://wormatlas.org**), a project headed by David Hall, which will provide an online encyclopedia of *C. elegans* cells and anatomy to construct CECAO with a top-down approach. In addition, we are also joining forces with other model organism databases (MODs) to come up with a set of shared controlled vocabularies.

Cell lineage and other aspects of development implicitly contain temporal information, e.g. because we know when each and every cell divides, we can know how many cells are there at a given time in development. Wen Chen has constructed a *C. elegans* life stage ontology that relates the total number of cells to defined life stages (personal communication). In the future, we will merge the life stage ontology with CECAO.

Lastly, CECAO will support extension to other nematodes. Many nematodes are known to also have mostly invariant anatomy and cell lineage; however, the lineages differ from those of *C. elegans* (e.g. [3,8,9,13]). CECAO can be extended to include comparative developmental information, and thus support queries across nematode species.

### References

1. Albertson DG, Thomson JN. 1976. The pharynx of *C. elegans*. *Phil Trans R Soc Lond B Biol Sci* **275**: 299–325.
2. Chitwood BG, Chitwood MB. 1974. *Introduction to Nematology (reprinted)*. University Park Press: Baltimore, MD; 334.
3. Felix MA, Sternberg PW. 1996. Symmetry breakage in the development of one-armed gonads in nematodes. *Development* **122**: 2129–2142.
4. Kimble J, Hirsh D. 1979. The postembryonic cell lineages of the hermaphrodite and male gonads in *Caenorhabditis elegans*. *Dev Biol* **70**: 396–417.
5. Newman A, White JG, Sternberg PW. 1996. Morphogenesis of the *C. elegans* hermaphrodite uterus. *Development* **122**: 3617–3626.
6. Riddle DL, Blumenthal T, Meyer BJ, Priess JR. 1997. *C. elegans II*. Cold Spring Harbor Laboratory Press: New York; 1–1222.

Copyright © 2003 John Wiley & Sons, Ltd.

7. Sharma-Kishore R, White JG, Southgate E, Podbilewicz B. 1999. Formation of the vulva in *Caenorhabditis elegans*: a paradigm for organogenesis. *Development* **126**: 691–699.
8. Sommer RJ, Sternberg PW. 1995. Evolution of cell lineage and pattern formation in the vulval equivalence group of *Rhabditid* nematodes. *Dev Biol* **167**: 61–74.
9. Sternberg PW, Horvitz HR. 1981. Gonadal cell lineages of the nematode *Panagrellus redivivus* and implications for evolution by the modification of cell lineage. *Dev Biol* **88**: 147–166.
10. Sulston JE. 1976. Post-embryonic development in the ventral cord of *C. elegans*. *Phil Trans R Soc Lond B Biol Sci* **275**: 287–298.
11. Sulston JE, Albertson DG, Thomson JN. 1980. The *C. elegans* male: postembryonic development of non-gonadal structures. *Dev Biol* **78**: 542–576.
12. Sulston JE, Horvitz HR. 1977. Post-embryonic cell lineages of the nematode, *Caenorhabditis elegans*. *Dev Biol* **56**: 110–156.
13. Sulston JE, Schierenberg E, White JG, Thomson JN. 1983. The embryonic cell lineage of the nematode *C. elegans*. *Dev Biol* **100**: 64–119.
14. Ward S, Thomson N, White JG, Brenner S. 1975. Electron microscopical reconstruction of the anterior sensory anatomy of the nematode *C. elegans*. *J Comp Neurol* **160**: 313–337.
15. White JG, Southgate E, Thomson JN, Brenner S. 1986. The structure of the nervous system of the nematode *C. elegans*. *Phil Trans R Soc Lond B Biol Sci* **314**: 1–340.
16. Wood WB and the Community of *C. elegans* Researchers. 1988. *The Nematode Caenorhabditis elegans*. Cold Spring Harbor Laboratory Press: New York; 1–667.

Copyright © 2003 John Wiley & Sons, Ltd. 
